# Bacterial community structure and bacterial isolates having antimicrobial potential in shrimp pond aquaculture

**DOI:** 10.1186/s13568-022-01423-9

**Published:** 2022-06-23

**Authors:** Sardar Ali, Jianmin Xie, Sahib Zada, Zhong Hu, Yueling Zhang, Runlin Cai, Hui Wang

**Affiliations:** 1 Biology Department, Institute of Marine Sciences, College of Science, Guangdong Provincial Key Laboratory of Marine Biotechnology, Shantou University, Shantou, 515063 China; 2 Southern Marine Science and Engineering Guangdong Laboratory (Guangzhou), Guangzhou, 511458 China

**Keywords:** Aquaculture, Pathogens, Shrimp ponds, Bacterial diversity, Metagenomics

## Abstract

Diseases outbreaks in pond aquaculture have resulted in huge losses to the aquaculture industry. The emergence of non-antimicrobial and environment friendly agents (probiotics) is the potential consideration for the healthy shrimp aquaculture. The present study was aimed to compare the bacterial community compositions in shrimp ponds and surrounding seawater, as well as isolate probiotic bacteria from the shrimp ponds. Based on the high-throughput of 16S rRNA gene sequencing, all sequences were assigned to 3584 unique operational taxonomic units (OTUs) at 97% similarity levels, which were affiliated with 24 phyla, 54 classes, 235 families, and 367 genera. The 10 most abundant phyla were Bacteroidota, Proteobacteria, Actinobacteriota, Planctomycetota, Cyanobacteria, Chloroflexi, Firmicutes, Desulfobacterota, Patescibacteria and Verrucomicrobiota. Notably, the alpha diversity (Shannon diversity) of shrimp ponds was significantly differences (*P* < 0.05) with that of surrounding seawater. There were 2498 and 791 unique OTUs in shrimp ponds and surrounding seawater, respectively. A total of 15 isolates were obtained in the culturable bacterial diversity, and the antibacterial activities were recorded for potential probiotic bacterial isolates against different tested bacterial isolates including pathogenic bacteria. An isolate *Hallobacillus* *marinus* HMALI004 showed strong inhibitory effects against three pathogenic bacteria, *Vibrio cholerae* CECT 514, non AHPND *V. parahaemolyticus* BCRC12959 and AHPND *V. parahaemolyticus* PD-2. The isolates *Algophigus* *sanaruensis* AGALI005, *Algoriphagus taiwanensis *ATALI009 and *Bacillus* *aequororis* BAALI008 were also identified as potential probiotics strains.

**Supplementary Information:**

The online version contains supplementary material available at 10.1186/s13568-022-01423-9.

## Introduction

Marine aquaculture is a fast-growing food industry, accounting about half of the total seafood supply (Garcia and Rosenberg 2010). In the production of mariculture between 1976 and 2016, 245% increase has been reported, which ultimately significantly enhance the exports from USD 8 billion (in 1976) to USD 143 billion in 2016, while in 2017 the demand values increased by 7% to peak at an estimated 152 billion USD (FAO 2018). On the mariculture, shrimp farming is one of the most important aquaculture industries, with a global record production of 512 kt worth USD 32 billion in 2016 (FAO 2018). Shrimps are usually cultivated in small seawater ponds (< 1 ha), which provide many advantages to farmers, including easy management, adjustable stocking density, and large production in a unit area (Li et al. 2010; Martınez-Cordova et al. 2015).

Nevertheless, emerging pathogens restrain the production of aquaculture affecting both economy and socio-economy of countries. There are various methods used so far to control the aquaculture diseases, such as, antibiotics and synthetic chemicals. Antimicrobial resistance (AMR) is considering the most serious problem to human health and animals. It is estimated that in 2050 around 10 million peoples will die from this resistant infection with associated economic cost of up to £60 trillion (O-Neill 2016). Around 70% of the antibiotics were used in livestock and this need of consumption is assumed to rise by 67% in 2030 (Van Boeckel et al. 2015). Till now this is a major concern since the overuse of antibiotics has been identified as the single most important factor pointing toward rise in resistant infections (CDC 2013).

Antibiotics are commonly used in larval cycle and in growth phases on aquaculture production. Antibiotic residues accumulated in the edible shrimp tissues that may alter intestinal flora of human and cause allergy and food poisoning problems (Thornber 2020). Most frequently used antibiotics to combat bacterial diseases include sarafloxacin, florfenicol, enrofloxacin and oxytetracycline (Roque et al. 2001; SotoRodríguez et al. 2006). Other antibiotics are also used globally such as ciprofloxacin, oxolinic acid, gentamicin, chlortetracycline, quinolones, norfloxacin, perfloxacin, tiamulinand and sulfamethazine (Holmstrom et al. 2003). Expensive chemotherapy for overcoming aquaculture diseases has been criticized for their more unsupportive effects. Such diseases controlling methods are ineffective against newly emerging pathogens in large aquaculture ponds. Therefore, substitutive methods are the demand of the present hour for the development of healthy microbial ecosystem in aquaculture for the maintenance of aquatic organism’s health (Liu et al. 2013). Disease outbreaks in shrimp aquaculture is the major challenge that causes huge economic losses (Hai 2015). The common infectious pathogenic bacteria in shrimp aquaculture are Gram-negative bacteria such as strains of *Pseudomonas, Yersinia, Vibrio, Flavobacterium*, and *Aeromonas*. These pathogenic bacteria cause furunculosis, enteric red mouth disease, vibriosis, septicemia, hemorrhage (Dawood et al. 2019; Hamid et al. 2017; Wanna et al. 2021; Wiklund 2016; Won et al. 2020; Zommiti et al. 2018). Bacteria that caused shrimp diseases have extracellular DNA encoding toxins to result in high mortality via peeling hepatopancreatic tissues, ultimately leading to the death of shrimp. This rate of mortality leads to considerable losses in economy and shrimp farming industry (Restrepo et al. 2021).

Probiotics are an emerging and promising alternative to prevent infectious diseases in shrimp aquaculture. Probiotic application is shrimp aquaculture has been reported to improve health (Mehrabi et al. 2018), growth (Gobi et al. 2018), and immunity of shrimp (Ramesh and Souissi 2018), as well as improve water quality and prevent diseases by modifying the microbial composition of water and sediment in the farm (Deng et al. 2018; Meidong et al. 2018). Although there are many studies on the beneficial effects of probiotics in shrimp aquaculture, however, the research about the native bacterial diversity as potential probiotics in the shrimp pond system is still under consideration. Therefore, the present study investigated the native bacterial diversity in shrimp aquaculture ponds through culture-dependent and culture-independent techniques and explored potential probiotic bacterial strains in shrimp ponds through cross-talk experiments.

## Materials and methods

### Samples collection and environmental parameters measurement

Water samples were collected from the ponds of four local shrimp farms (Pond 1 = P1, Pond 2 = P2, Pond 3 = P3, Pond 4 = P4) and (Surrounding seawater = S) in Shantou, Guangdong province, Southern China (23.36°N, 116.66°E) on September 2019. All four ponds are adjacent to each other at a distance of 115–140 m, and separated by surrounding seawater at a distance of 970 m. The length, width, and depth of the ponds were about 200, 60, and 1.5 m, respectively (Additional file 1: Table S3). All water samples were collected three three different locations of each pond and surrounding seawater from a depth of about 28 cm and stored in sterile polycarbonate bottles (Additional file 1: Fig. S2). Samples were kept at 4 °C and transferred to the laboratory for further analysis within 12 h. Water samples were subsampled for further investigation. Physicochemical parameters such as pH, temperature, and oxidation-reduction potential (ORP), were measured through HQ 30d multi-parameter water quality analyzer (HACH, Loveland, co). Salinity was measured by portable refractometer (Suwei, Guangzhou, China). Chemical oxygen demand (COD) was measured by using the dichromate method (Dedkov et al. 2000), and ammonia and nitrite were measured as described by Lu et al. (2009). Subsamples for DNA extraction were filtered through a nucleopore filter (diameter of 0.45 μm) and the filters were kept at − 80 °C until DNA extraction.

### Analysis of bacterial communities using high throughput sequencing

To study the bacterial community structure in shrimp ponds and surrounding seawater, environmental whole genome DNA was extracted from the filter membranes using a PowerSoil DNA Extraction Kit (MoBio Laboratories, Solana Beach, CA, USA) according to the manufacturer’s instruction. Bacterial communities were characterized at each time point of the incubation at 515 F and 907R primers (Raina et al. 2022), which mainly target the V4-V5 region of the bacterial 16S rRNA gene, which were used for PCR amplification of extracted DNA. PCR reaction included 6 µL of template, 2.5 µmol of each deoxyribonucleotide triphosphate (Bioline), 1 µL of UltraPure Bovine Serum Albumin (Thermo Fisher), 0.25 µL of Velocity DNA polymerase and 5 × PCR buffer (Bioline), 10 pmol of each primer (resuspended in UV sterilized water) with the following adaptors (5′^/^-GTGCCAGCMGCCGCGG-515 F-3^/^) and (5^/^-CCGTCAATTCMTTTRAGT-907R-3^/^). PCR programmed condition were 98 °C for 2 min, followed by 30 cycles of denaturation at 98 °C for 30 s, annealing for 30 Sect. (46 °C for 3 cycles, 48 °C for 3 cycles, and 50 °C for 24 cycles), 72 °C for 30 s, and final extension at 72 °C for 10 min. PCR clean-up, indexing and sequencing (llumina MiSeq (2 × 300 bp)) were performed at the BGI-Shenzhen (The Beijing Genomics Institute, Shenzhen, Guangdong, China) for sequencing.

After obtaining raw sequencing data, the barcodes were removed, and the primer and adapter sequence reading and metadata were imported into QIIME 2 and analyzed according to the tutorial (Caporaso et al. 2010). Briefly, the DADA2 algorithm (Callahan et al. 2016) was used for demultiplexing, denoising, truncating reads, and joining forward and reversed read pairs. Chimeras were identified using the VSEARCH ‘uchime_denovo’ method (Rognes et al. 2016) and subsequently removed. Respective reads were then summarized in a feature table. Features were then divided to the genus level, and a reduced table containing only highly abundant taxa (> 1% mean relative abundance) was used for generating bar charts.

Shannon-Wiener diversity index and phylogenetic diversity index in each sample were calculated using qiime2 (v2019.10.0) with diversity plugin, and their significance was measured by Kruskal-Wallis test. Microbial beta-diversity was determined by non-metric multidimensional scaling (NMDS) using a Bray–Curtis distance matrix between the samples based on the genus level, which was performed using an R module called ggvegan (v2.5-7).

## Isolation and identification of culturable bacteria

Culturable bacteria were isolated from shrimp ponds and surrounding seawater via serial dilution and cultural technique using marine agar 2216E plates (peptone 5.0 g, magnesium chloride 8.8 g, ferric citrate 0.1 g, potassium chloride 0.55 g, yeast extract 1.0 g, calcium chloride 1.8 g, sodium sulfate 3.24 g, sodium bicarbonate 0.16 g, potassium bromide 0.08 g, agar 15.0 g, sodium chloride 19.54 g, strontium chloride 34.0 mg, boric acid 22.0 mg, sodium fluoride 2.4 mg, sodium silicate 4.0 mg, ammonium nitrate 1.6 mg, disodium phosphate 8.0 mg in one liter of purified water). The most dominant and morphologically distinct colonies (in terms of size, shape, colour, fast-growing) were isolated, sub-cultured, and purified on marine agar 2216E plates. Pure colonies were preserved in 60% glycerol and 40% marine broth (v/v) and preserved at − 80 °C.

Moreover, DNA was extracted from purified bacterial colonies by using Chelex and proteinase K as lyzing agents. Briefly, aliquots (a spot) of bacterial colonies were added into 50 µL DNAse free water with 5% Chelex and 2.5 µL of proteinase K (20 mg/ml), and further incubated at 37 °C for 30 min to release DNA. The 16 S rRNA gene was amplified using bacterial specific universal primers 27 F (5′-AGAGTTTGATCCTGGCTCAG-3′) and 1492R (5′-TGACTGACTGAGGYTACCTTGTTACGACTT-3′) (Wang et al. 2012). The PCR mixture composed of 1.25 µL of 50 mM MgCl_2_, 2.5 µL of 10x PCR buffer, 0.2 µL of 25 mM dNTPs, 0.25 µL of Taq DNA polymerase (5 U/µL), 0.4 µL of each primer (25 pmol/µL), and 1 µL of template DNA. The final volume was implemented to 25 µL with DNAse free water. The amplification was carried out in a thermocycler (Mastercycler Personal 5332) under the following conditions, initial denaturation at 95 °C for 2 min followed by 30 cycles of denaturation at 95 °C for 90 s, annealing at 52 °C for 1 min and extension at 72 °C for 90 s; and a final extension at 72 °C for 10 min. The amplified products were then subjected to 1% agarose gel electrophoresis with ethidium bromide as a stain and visualized on a transilluminator under UV light.

Furthermore, the amplified 16S rRNA gene was sequenced by BGI-Guangzhou (The Beijing Genomics Institute, Guangzhou, Guangdong, China). ContigExpress (http://www.contigexpress.com/index.html) was used to assembled the sequences of 16S rRNA gene. Sequences were identified by comparing with the closest relatives in the GenBank database using BLAST (Basic Local Alignment Search Tool) (Additional file 1: Table S2). 16S rRNA gene sequences of representative genus from the families Moraxellaceae, Aeromonadaceae, Bacillaceae, Cyclobacteriaceae, Vibrionaceae, Nocardioidaceae, Erythrobacteraceae, and Shewanellaceae were downloaded and used to construct Neighbor-joining phylogenetic tree through MEGA-X. *Halobacterium salinarum* JCM 8978 (AB663362) was used as an outgroup. Bootstrap support is shown for cases where the value was greater than 50% based on 1000 replications (Ali et al. 2021). The scale bar indicates 0.01 substitution per nucleotide position. And GeneBank accession numbers were listed after each sequence name (Fig. 3). The major strains used in this study were deposited in China Center of Industrial Culture Collection (CICC).

**Fig. 1:**
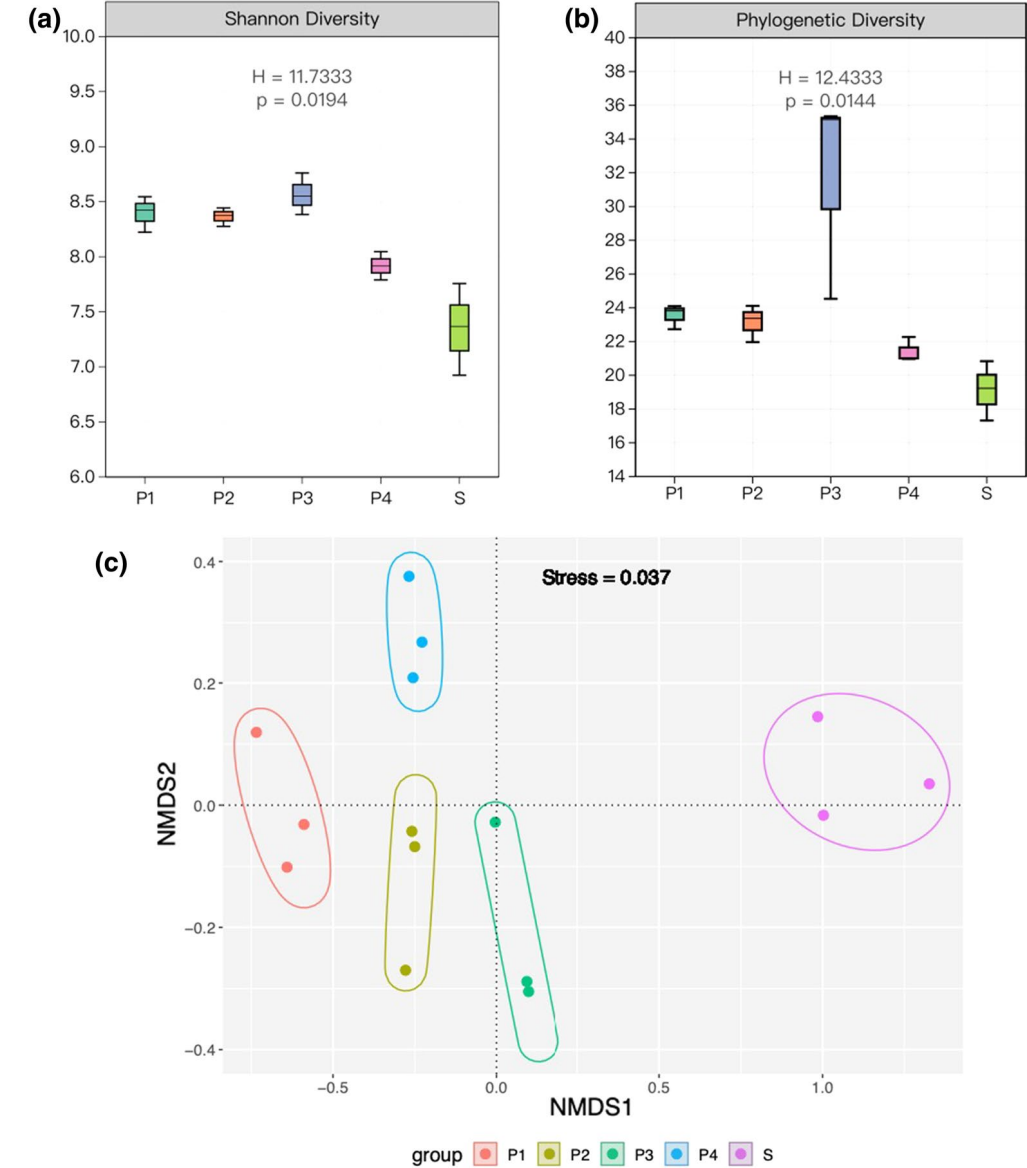
Significant differences (*P* < 0.05) were detected between alpha-bacterial communities (Shannon index) of surrounding seawater samples and shrimp ponds (**a**, **b**). Data was also clustered to Non-metric multidimensional scaling (NMDS) plot for beta-diversity (**c**)

### Cross-talk experiment to check the antimicrobial activity

To analyze the binary interaction between isolates, the stationary phase culture of each strain was tested for sensitivity against the other 15 strains. The 15 cryopreserved strains isolated in this study were re-cultured in marine broth for 24 h, with lawn as the target strain. The inhibitory activity of the strains was performed in Petri plates containing marine agar medium 2216E. About 50–100 µL of bacterial inoculum was spread uniformly on a sterile marine agar plate using a sterile cotton swab. A full loop of growing bacteria was placed on the surface of the lawn of the target bacteria and was incubated at 25 °C temperature for 24 to 72 h. The inhibition of the pathogenic bacterial growth was measured according to the size of the inhibition zone (Smania et al. 1999). Pathogenic *Vibrio* strains like non AHPND *V. parahaemolyticus* BCRC12959 and *V. parahaemolyticus* PD-2 were received from shrimp disease and immunity Lab, Collage of Science, Shantou University.

### Statistical analysis

The differences between shrimp ponds and surrounding seawater were analyzed by the Kruskal-Wallis test (also called one-way ANOVA on ranks) to check the significance and the *P* value < 0.05 was considered significant.

## Results

### Physicochemical characteristics of water samples

The physicochemical parameters of water in shrimp ponds and surrounding seawater varied in chemical oxygen demand (COD), oxidation-reduction potential (ORP), salinity, and ammonia (NH_4_) concentrations, while slight differences were detected for pH value. The ORP of shrimp ponds varied from 39 mv to 86 mv, as compared to the surrounding seawater which was 71 mv. The salt concentration was low in all four ponds ranging from 0.5 to 0.9%, compared to seawater samples recorded 1%. The COD level in different shrimp ponds varied from 35.3 mg/L to 45.4 mg/L, while the value for the seawater sample was 55.5 mg/L. The ammonia (NH_4_) concentrations in different shrimp ponds ranged from 2.2 mg/L to 3.7 mg/L, and the value for seawater was 2.2 mg/L. The observed pH of different shrimp ponds varied from 7.7 to 8.5, while the value for the seawater sample was 7.2. The temperature of different shrimp ponds was in the range of 28.3 to 29.5 °C, while for seawater samples was 29.5 °C (Additional file 1: Table S1).

### Composition of bacterial communities in shrimp ponds and surrounding seawater

Through Illumina high throughput sequencing analysis of bacterial diversity in the shrimp ponds and surrounding seawater, a total of 762,863 sequences were obtained. The range of effective reads in the water samples was from 50,568 to 51,275. All sequences were assigned to 3584 unique OTUs, belonging to 24 phyla, 54 classes, 235 families, and 367 genera. Significant differences (*P* < 0.05) were detected between alpha-bacterial communities (Shannon index) of seawater samples and shrimp ponds (Fig. 1a). Pond 3 (P3) showed substantial phylogenetic diversity compared to other samples (Fig. 1b). Interestingly, P1 and P2 showed similarities in bacterial diversity to P4 while distinct from surrounding seawater samples (S) (Fig. 1b). Data was also clustered to NMDS plot for beta-diversity (Fig. 1c), in which bacterial diversity of shrimp ponds was significantly different from those in the surrounding seawater.

**Fig. 2:**
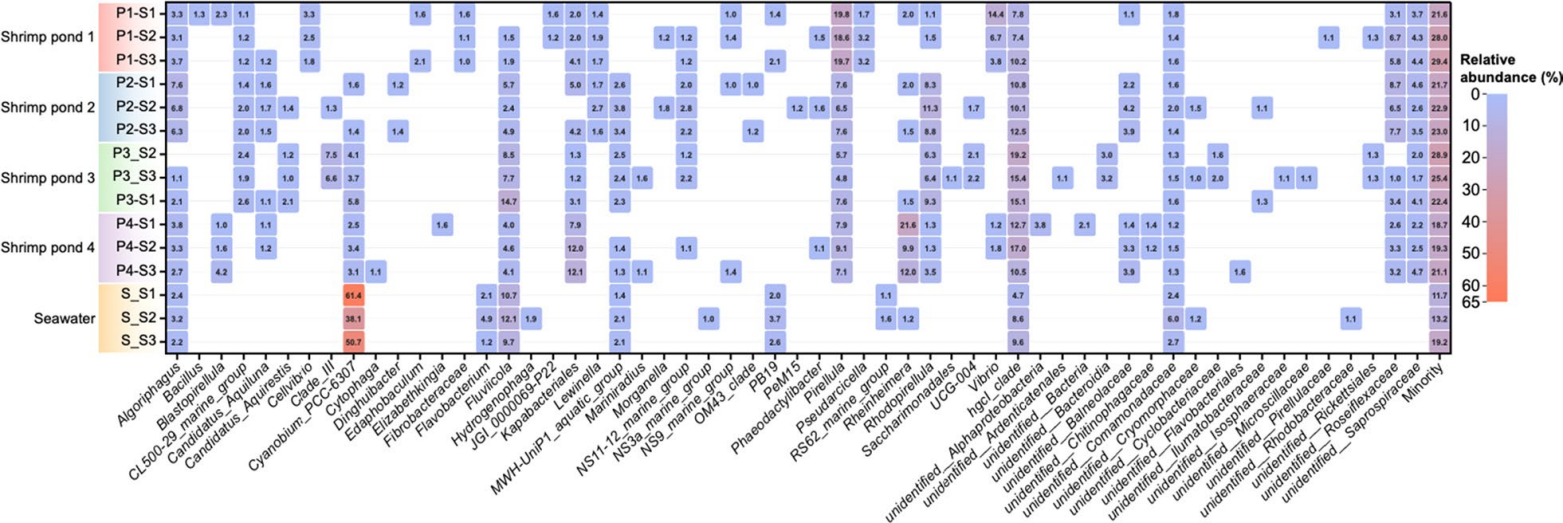
Heatmap showing relative abundance and community compositions of bacterial genera in shrimp ponds and seawater samples. The genus-level distribution is based on the 97% similarity clusters OTUs. Sequences whose relative abundance was lower than 1% were assigned as “Minority”

Notably, few bacterial phyla (Bacteriodetes, Proteobacteria, Actinobacteria, Planctomycetota, Cyanobacteria, Chloroflexi, and some other phyla with low concentration) were detected in both shrimp ponds and the surrounding seawater. In shrimp ponds, the major bacterial phyla were Bacteriodetes (27.5–35.4%), Proteobacteria (12.5–61.4%), Actinobacteria (13.5–22.2%), Planctomycetota (12.8–23.1%), while Cyanobacteria (50.45%) were the dominant phyla in surrounding seawater (Additional file 1: Fig. S1).

The genus-level bacterial community structure of shrimp ponds and surrounding seawater is summarized in Fig. 2. Bacterial community in shrimp ponds was diverse than the surrounding seawater community in terms of composition and their relative abundance. The most abundant bacterial genera in shrimp ponds were *Pirellula*, while *Cyanobium* was the dominant bacterial community in surrounding seawater. The relative abundance of *Pirellula* in shrimp ponds ranged from 18.1 to 58%, but it was not detected in surrounding seawater. In shrimp ponds, the relative abundance of hgcl-clade bacteria (8.4–16.5%) was significantly higher (*P* < 0.05) than that in surrounding seawater (7.6%). The relative abundance of genus *Cyanobium* in surrounding seawater (52.6%) was significantly higher (*P* < 0.05) than that in shrimp ponds (0.3–4.5%). The relative abundance of *Rhodopirellula* in shrimp ponds was 1.4–9.4%, while it was not found in surrounding seawater. In shrimp ponds, the relative abundance of unidentified *Kapabacteria* from phylum Bacteroidetes was 1.8–10.6%, but it was not detected in surrounding seawater. The relative abundance of unidentified Roseiflexaceae, belonging to phylum Chloroflexi, in shrimp ponds (5.4–23%) was significantly higher (*P* < 0.05) than surrounding seawater (1.3%).

**Fig. 3:**
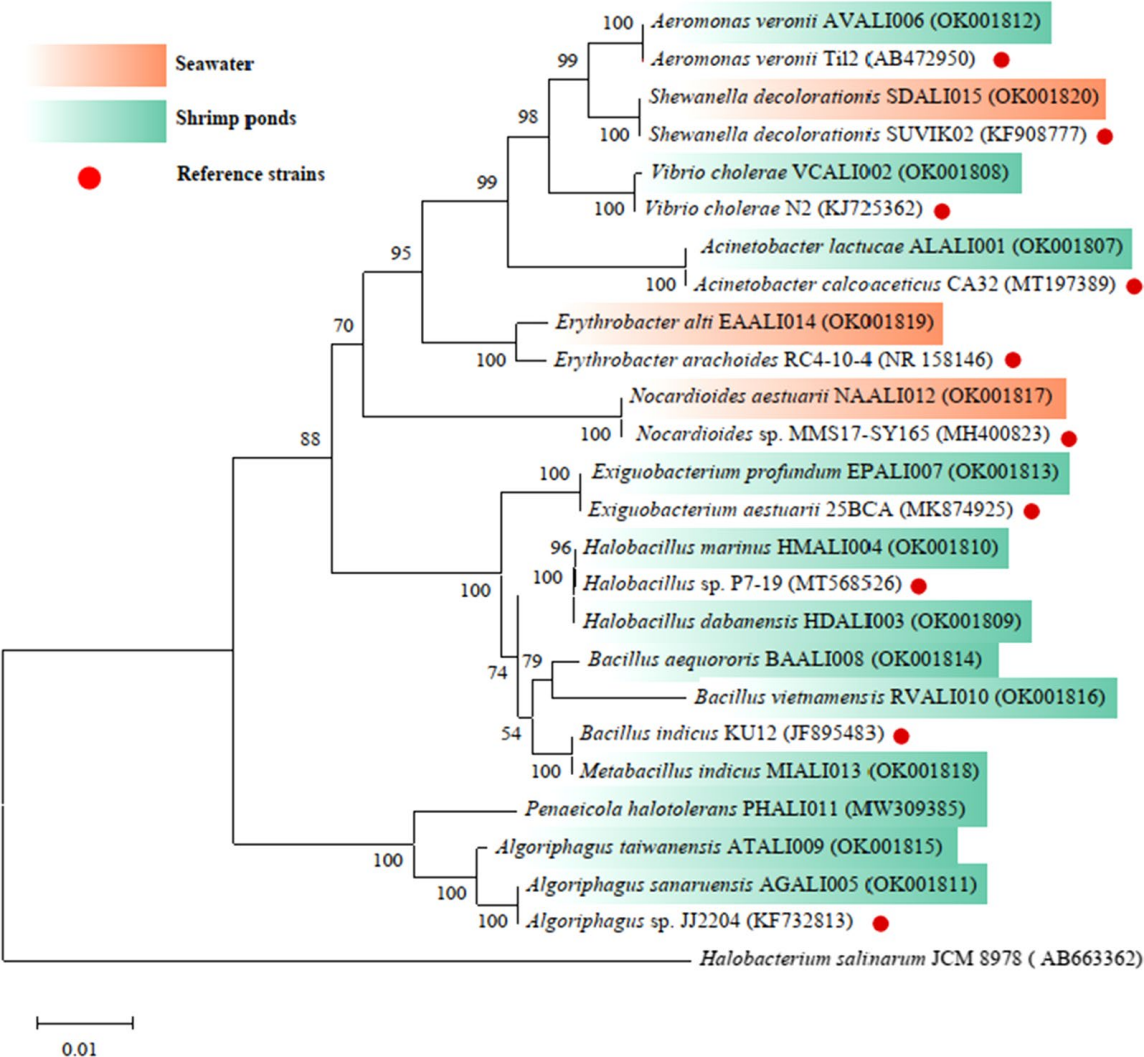
Neighbor-joining phylogenetic tree based on 16S rRNA gene sequences showing the phylogenetic position of all isolated strains. *Halobacterium salinarum* JCM 8978 (AB663362) was used as the outgroup. Bootstrap support is shown for cases where the value was greater than 50% based on 1000 replications. The scale bar indicates 0.01 substitution per nucleotide position. Strains from the shrimp ponds were highlighted as green shade, and the strains from surrounding seawater were highlighted as orange shade, while the reference strains were marked with red dots, and GeneBank accession numbers listed after each sequence name

The genus *Algoriphagus* in shrimp ponds was found (1.4–6.9%) abundant than surrounding seawater (2.5%). *Rheinheimera* from the family Alteromonadaceae in shrimp ponds ranged from 1.3 to 14.4%, however, the relative abundance of this genus in the surrounding seawater sample was 1.9%. The relative abundance of unidentified Saprospiraceae in shrimp ponds (1.7–3.3%) was significantly higher (*P* < 0.05) than that in surrounding seawater (0.2%). In addition, the abundance of *Bacillus* in shrimp ponds ranged from 0.03 to 0.3%, but it was not detected in surrounding seawater. The relative abundance of unidentified Comamonadaceae in shrimp ponds (0.9–1.6%) was significantly lower (*P* < 0.05) than that in surrounding water (3.7%). Furthermore, the pathogenic bacteria *V. cholera* was identified in shrimp pond 1 (P1).

### Isolation of bacterial strains

Bacterial strains were isolated and 15 strains were selected based on their distinct morphology. The findings of 16S  rRNA gene sequence indicated that the bacterial isolates from shrimp ponds were *Bacillus* (3 strains), *Algoriphagus* (3 strains), *Vibrio* (1 strain), *Aeromonas* (1 strain), *Acinetobacter* (1 strain), *Exiguobacterium* (1 strain), and *Halobacillus* (2 strains). In contrast, *Nocardioides* (1 strain), *Erthrobacter* (1 strain), and *Shewanella* (1 strain) were found to be the dominant genera in surrounding seawater (Fig. 3). In the phylogenetic tree, strains from the shrimp ponds were highlighted as green shade, and the strains from surrounding sea water were highlighted as orange shade, while the reference strains were marked with red dots (Fig. 3). Detailed information of the BLAST results of individual strains were provided in (Additional file 1: Table S2).

### Candidate probiotics strain through cross-talk

**Fig. 4:**
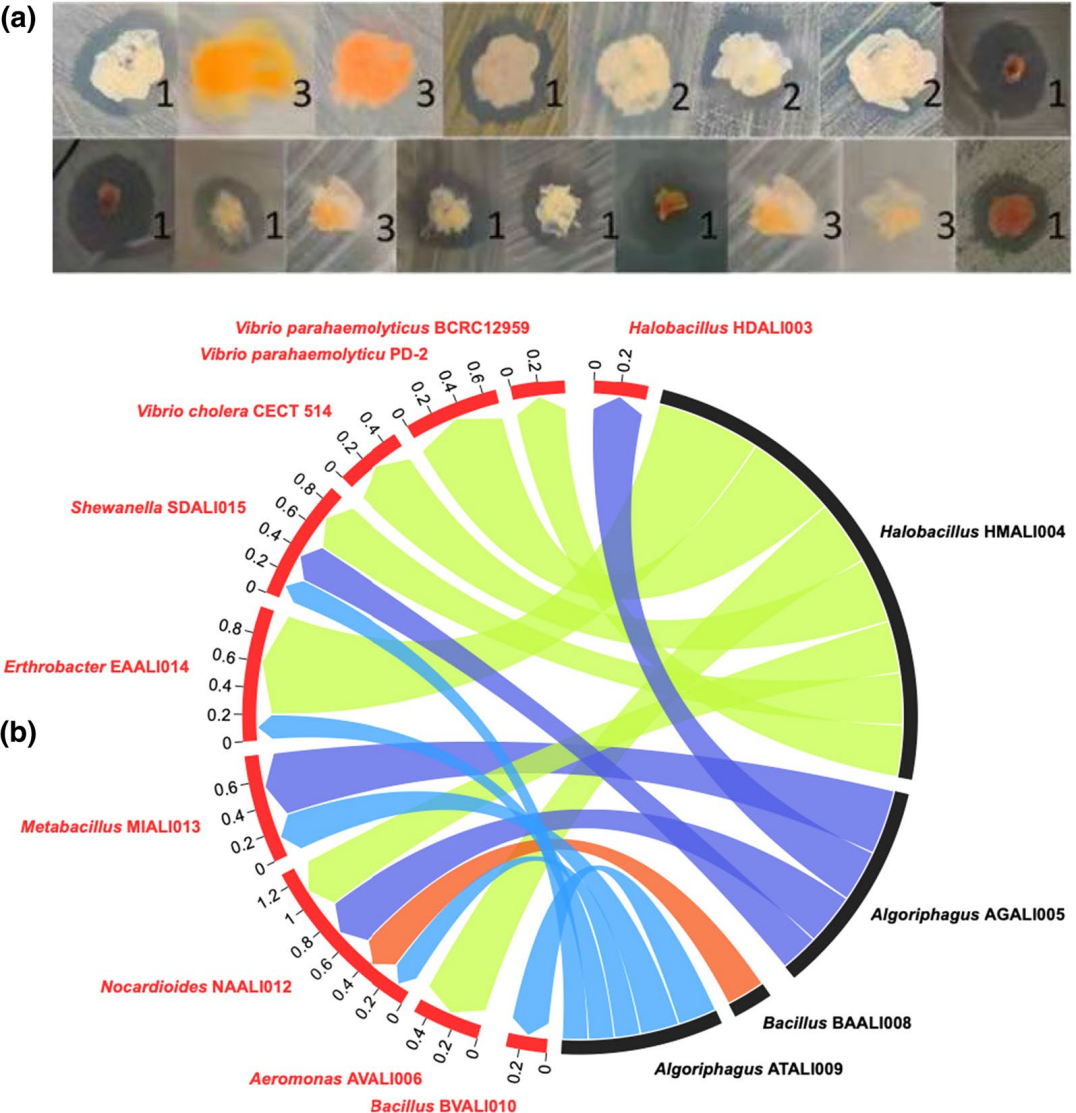
Inhibitory activity (**a**) and Chord-diagram (**b**), Inhibitory zone in **a** is labeled with 1 for strong, 2 for weak, and 3 for no inhibition based on halo size. In Chord-diagram the targeted strains (inhabited) marked in red color, whereas strains with probiotic activity was highlighted in black color. The findings show that probiotics bacterial isolates such as *H. marinus* HMALI004, *B. aequororis* BAALI008, *A. sanaruensis* AGALI005 and *A. taiwanensis* ATALI009 inhabited the growth of *N. aestuarii* NAALI012, *E.* alti EAALI014, *S.* *decolorationis* SDALI015, *B. vietnamensi* BVALI010, *H. dabanensis* HDALI003, *V. parahaemolyticus* PD-2, *V. cholerae* CECT 514T, and non AHPND *V. parahaemolyticus* BCRC12959. The number at the end of nodes represents the inhibitory values in milimeter

To test the inhibitory interaction among 15 bacterial isolates, each isolate was tested for potential growth inhibition against all other isolates. The results indicated that more than 225 pairings of the 15 isolates were monitored for pathogenicity in shrimp ponds. Notably, 17 (15 were isolated via culturable method and 2 were collected from shrimp disease and immunity Lab, Collage of Science, Shantou University, Shantou Guangdong China) strains showed binary inhibition (Fig. 4a). Four stains showed inhibitory binary interaction (Fig. 4b). Most of the observed inhibition was due to the activity of strains in two bacterial phyla, Bacteriodetes and Firmicutes. Strain ATALI009 (CICC accession number 25,154) showed the highest 16S rRNA gene similarity with *Algoriphagus taiwanensis* CC-PR82 (99.4%), and inhibited the growth of strains BVALI010 (*Bacillus vietnamensis)*, NAALI012 (*Nocardioides aestuarii*) (pathogenic), SDALI015 (*Shewanella decolorationis*) (pathogenic), and EAALI014 (*Erthrobacter alti*) (pathogenic), with an inhibition zone of 0.3 mm, 0.2 mm, 0.3 mm, and 0.2 mm, respectively. Strain AGALI005 (CICC accession number 25,153) which is also grouped into the genus of *Algoriphagus* (99.1% similarity with *Algoriphagus sanaruensis* M8-2), could inhibit the growth of strains HDALI003 (*Halobacillus dabanensis)*, NAALI012 (*N. aestuarii)*, BVALI010 (*B. vietnamensis*), and SDALI015 (*S. decolorationis)* by 0.4 mm, 0.4 mm, 0.5 mm, and 0.3 mm, respectively. Strain BAALI008 (CICC accession number 25,152) belongs to the genus *Bacillus* (alternative of *Sutcliffiella* showed 99.7% similarity with *Bacillus horikoshii* M-8), which could inhibit the growth of strain NAALI012 (*N. aestuarii)*, with an inhibition zone of 0.3 mm. HMALI004 (*H. marinus*) (CICC accession number 25,155) inhibited the growth of strains AVALI006 (*Aeromonas veronii*), NAALI012 (*N. aestuarii*), EAALI014 (*E. alti*), and SDALI015 (*S. decolorationis*), with inhibition zones of 0.5 mm, 0.4 mm, 0.8 mm, and 0.4 mm, respectively. Strain HMALI004 with the highest similarity to *H. marinus* KGW1 (99.7%) was further applied to assess the inhibitory potential against well-studied bacterial marine pathogens, including *V. cholerae* CECT 514T, *V. parahaemolyticus* PD-2, and non AHPND *V. parahaemolyticus* BCRC12959. Strain HMALI004 showed dramatic inhibitory activity against all tested model pathogens, and the inhibiting zones of CECT 514, PD-2, and BCRC12959 were 0.5 mm, 0.7 mm, and 0.4 mm against strains, respectively (Fig. 4a).

## Discussion

Microorganisms are essential in aquatic environments due to their potential to degrade pollutants, improve water quality and prevent the growth of pathogens (Martinez-Cordova et al. 2015). In addition, they play a pivotal role in the health of aquatic life and the sustainability of aquaculture ecosystems (Ninawe and Selvin 2009), the microbial diversity of the shrimp ponds system remains to be studied (Alfiansah et al. 2018; Hastutia et al. 2021; Martinez-Porchas and Vargas-Albores 2017; Zeng et al. 2020; Zhang et al. 2019).

In the present study, the relative abundance of Bacteriodetes, Proteobacteria, Actinobacteria, Planctomycetota, and Chloroflexi in shrimp ponds were significantly higher than in surrounding seawater, while the relative abundance of Cyanobacteria in surrounding seawater was significantly higher than that in shrimp ponds. These findings are consistent with a report that Proteobacteria, Bacteroidetes, and Actinobacteria were the leading phyla of pond aquaculture (Zheng et al. 2017, 2021). However, it contradicted with the results of Dabade et al. (2016), in which Proteobacteria, Flavobacteria, and Actinobacteria were the major phyla in shrimp aquaculture ponds. This abundance of microbial communities in our study might be due to similar condition of water in all shrimp ponds.

Genus hgcl-clade belongs to Actinobacteria, and its relative abundance in shrimp ponds was very high. This bacterium has been reported to be a potential probiotic in aquaculture ponds (Das et al. 2008). Actinobacteria possess the potential of probiotics to improve the digestibility and immune system of shrimp to counteract the pathogenicity of pathogens (Costantini et al. 2017). Genus *Rhodopirellula* belonging to Actinobacteria was observed in ponds water, while it was not found in surrounding seawater. Genomic analysis of *Rhodopirellula* sp. revealed the stigmatellin, myxothiazol, and myxamid antibiotics encoding genes (Huntley et al. 2012). This group of bacteria could be a potential probiotic in shrimp ponds. Another dominant bacteria in shrimp ponds was *Rheinheimera* sp., which was previously isolated from the rhizosphere of *Echinacea purpurea* (Carolina et al. 2019). Comamonadaceae is an aerobic organotrophs, Fe^3+^ reducing and anaerobic denitrifiers, hydrogen oxidizers, photoheterotrophic and photoautotrophic, and fermentative bacterial family. Most of the members of this family are environmental bacteria reported from water and soil habitats. Some members of the Comamonadaceae family are also probiotics (Willems 2014; Qin et al. 2016) studied the abundance and diversity of bacteria in pond water augmented with various feeds and found that the ponds supplied with sudangrass had better water conditions. Moreover, in the ponds supplied with sudangrass, pathogenic bacteria such as *Acinetobacter* and *Aeromonas* decreased significantly, while probiotic unclassified Comamonadaceae increased significantly. In this study, the relative abundance of Saprospiraceae sp. in shrimp ponds water was significantly higher than that in surrounding seawater. This species was mostly reported from marine water as predators of other bacteria and microalgae (Mcllroy and Nielsen 2014). Saprospiraceae sp. showed activity against the marine pathogen *V*. *shiloi* (Choi et al. 2015). The bacterial population in shrimp ponds is the key to protect the shrimp pond ecosystem from pathogens (Alfiansah et al. 2018).

The bacterial diversity in shrimp ponds was significantly higher than that in surrounding water. Since a high microbial community is important for the stability of ponds and the digestion and immunity of shrimp, the diverse microbial community in shrimp ponds could improve the quality of water and shrimp health (Fan et al. 2019; Zhang et al. 2016). In the current study, four bacterial strains with antimicrobial activities were isolated through culturable method, and two strains were affiliated to Firmicutes (*H. marinus* HMALI004 and *B. aequororis* BAALI008), and the other two strains were affiliated to Bacteroidetes phylum (*A. sanaruensis* AGALI005 and *A. taiwanensis* ATALI009). Except for *H. marinus* HMALI004, the other three were found in 16S rRNA high-throughput sequencing. These bacterial strains act as probiotics in shrimp ponds. Jang et al. (2019) used *Bacillus* sp. as a dietary probiotic supplement in the feed of olive flounder to fight against the pathogenic challenge and found a significant abundance of *Bacillus* sp. in the intestine after 14 days, which enhanced the richness of the bacterial community, immune response, digestive enzymes activities, and protection from pathogens. Thus, the use of probiotic bacteria *Bacillus* sp. in aquaculture has become the best strategy to prevent pathogens and increase nutrient assimilation.

Bacteria affiliated with the genus of *H. marinus* HMALI004 were isolated from shrimp ponds. Through cross-talk experiments, this bacterial strain could inhibit the growth of *Nocardioides, Erthrobacter*, and *Shewanella*, which have been proved to be pathogens in aquaculture ponds (Mehta and Shamoo 2020; Tseng et al. 2018). Moreover, *H. marinus* HMALI004 also inhibited the growth of well-studied marine pathogens, including *V*. *parahaemolyticus* PD-2, *V*. *cholerae* CECT 514T, and non AHPND *V*. *parahaemolyticus* BCRC12959. Similar results were recorded by Mayavu et al. (2014), in which *Hallobacillus* isolated from parangipettai salt pan environment inhibited the growth of *V*. *angullaram*, *V. paraheamolyticus*, and *V*. *alginolyticus.* Likewise, based on the antagonistic activity against shrimp pathogens, AshokKumar and Mayavu (2014) isolated 25 halobacterial strains from the Marakanam salt pan environment, and among all 25 bacteria only one bacterial strain, *Bacillus* Mk22 showed high antagonistic activities. Recently, the use of halophilic bacterial applications has increased in industries. It secretes a wide range of various bioactive metabolites, such as enzymes (amylase, protease, cellulases, etc.), extra-cellular polysaccharides (EPS), proteins, etc. (Enache et al. 2010).

Bacteria affiliated with the genus of *A. taiwanensis* ATALI009 were isolated from shrimp ponds, and OTUs in this genus were also detected in 16S rRNA high throughput sequencing. Through cross-talk experiments, these bacterial strains could inhibit the growth of *Nocardioides, Shewanella*, and *Erthrobacter*, which have been proved to be pathogens in aquaculture ponds (Mehta and Shamoo 2020; Tseng et al. 2018). *Algoriphagus* sp. has a carotenoid gene cluster (*ispH*, *crtYcd*), which plays a vital role in photosynthesis, protection against oxidative damage, and nutrition (Tao et al. 2006). Carotenoids are pigments that may convert to vitamin A, and it can also act as an antioxidant (Sajjad et al. 2020) to protect shrimps from infection by strengthening the immune system (Wilding et al. 2000). The present study provides the first insight in the role of *Algoriphagus* as a probiotic in pond culture. In conclusion, the bacterial community in Shantou shrimp ponds was dominated by Proteobacteria, Firmicutes, Bacteriodetes, Actinobacteria, and Planctomycetes. Through unculture method, 16S rRNA high throughput sequencing, probiotic bacteria isolated from shrimp ponds were genus hgcl-clade, *Rhodopirellula*, *Rheinheimera* sp, Comamonadaceae, Saprospiraceae sp. Four probiotic bacterial strains such as *H. marinus* HMALI004, *B. aequororis* BAALI008, *A. sanaruensis* AGALI005 and *A. taiwanensis* ATALI009 were successfully isolated through culture dependent method luckily the OTUs for these strains were also present in unculturable method, some of which were first case reports of the species. Our study revealed that a novel source of antimicrobial metabolites from these probiotic strains could manage and reduce the risk of pathogen that causes shrimp diseases. Continuing the current research work in the future, the metabolites will be extracted and studied further for antimicrobial activities against various pathogenic bacterial strains. These findings provide insights to better understand the role of probiotic bacteria in shrimp ponds.

## Supplementary information

**Additional file 1: FigureS1: **Phylum level relative abundance and communitycompositions of shrimp ponds and surrounding sea water samples obtained by 16S rRNA High through put-sequencing in 15 samples. The phylum level distributionis based on the 97% similarity clusters OTUs. Sequences whose relativeabundance was lower than 1% were assigned as “Minority”. **Figure S2: **Actual figures of sampling locations. **TableS1. **Psychochemical parameters of shrimp ponds and surrounding sea water.**Table S2. **BLAST results of the isolated stains.** Table S3.** Details ofselected sampling area.

## Data Availability

All raw reads of 16S rRNA gene based High throughput sequencing were deposited in the sequencing read archive (SRA) of NCBI for diversity analysis with accession numbers from SAMN20970002 to SAMN20970016 under the Bioproject number-PRJNA757637. 16S rRNA gene sequences of all bacterial isolates were deposited to NCBI GenBank (accession numbers: OK001807- OK001820).
