# Supplementary material for: Bacterial community structure and bacterial isolates having antimicrobial potential in shrimp pond aquaculture
Source: AMB Express. 2022 Jun 23;12:82. doi: 10.1186/s13568-022-01423-9 (PMC9226248; doi:10.1186/s13568-022-01423-9)
Supplement: Supplementary file 1 — Additional file 1: FigureS1: Phylum level relative abundance and communitycompositions of shrimp ponds and surrounding sea water samples obtained by 16S rRNA High through put-sequencing in 15 samples. The phylum level distributionis based on the 97% similarity clusters OTUs. Sequences whose relativeabundance was lower than 1% were assigned as “Minority”. Figure S2: Actual figures of sampling locations. TableS1. Psychochemical parameters of shrimp ponds and surrounding sea water.Table S2. BLAST results of the isolated stains. Table S3. Details ofselected sampling area. [file 13568_2022_1423_MOESM1_ESM.pdf]

AMB Express

Supplementary Materials

Bacterial community structure and bacterial isolates having antimicrobial potential in shrimp pond aquaculture

Sardar Ali[1, #], Jianmin Xie[1, #], Sahib Zada[1], Zhong Hu[1, 2], Yueling Zhang[1, 2], Runlin Cai[1, *], Hui Wang[1, 2, *]

[1] Biology Department and Institute of Marine Sciences, College of Science, and Guangdong Provincial Key Laboratory of Marine Biotechnology, Shantou University, Shantou, China 515063

[2] Southern Marine Science and Engineering Guangdong Laboratory (Guangzhou), Guangzhou, China, 511458

Corresponding author: Hui Wang, wanghui@stu.edu.cn, +86-754-86502721; Runlin Cai, rlcai@stu.edu.cn, +86-754-86502721.

[#]These authors contributed equally.

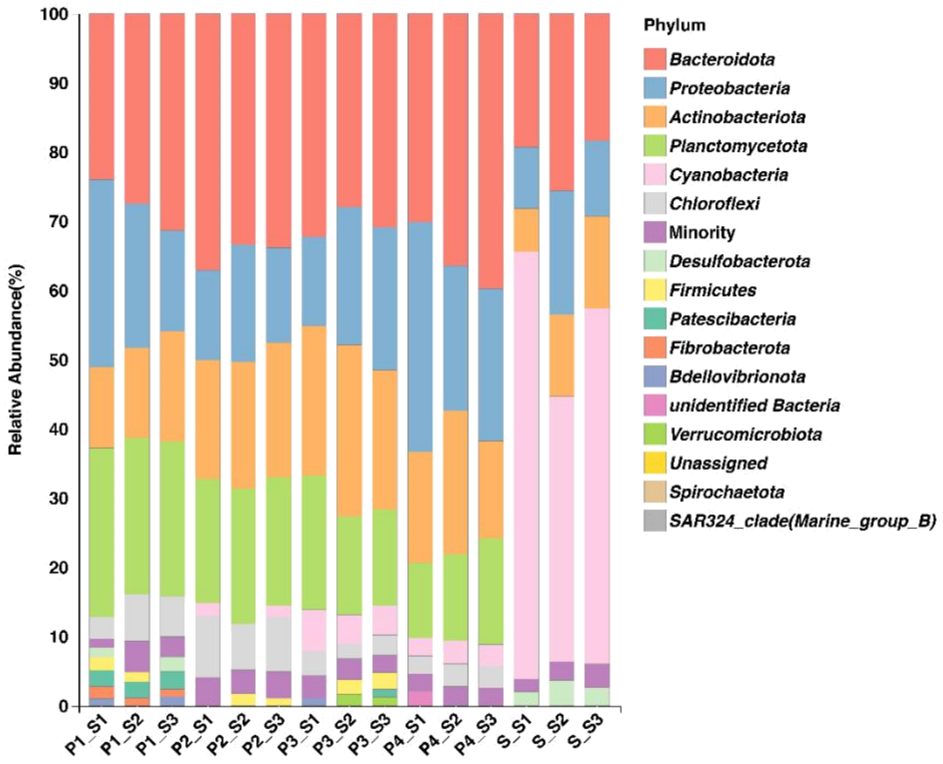

Supplementary Figure S1: Phylum level relative abundance and community compositions of shrimp ponds and surrounding sea water samples obtained by 16S rRNA High through put-sequencing in 15 samples. The phylum level distribution is based on the 97% similarity clusters OTUs. Sequences whose relative abundance was lower than 1% were assigned as “Minority”.

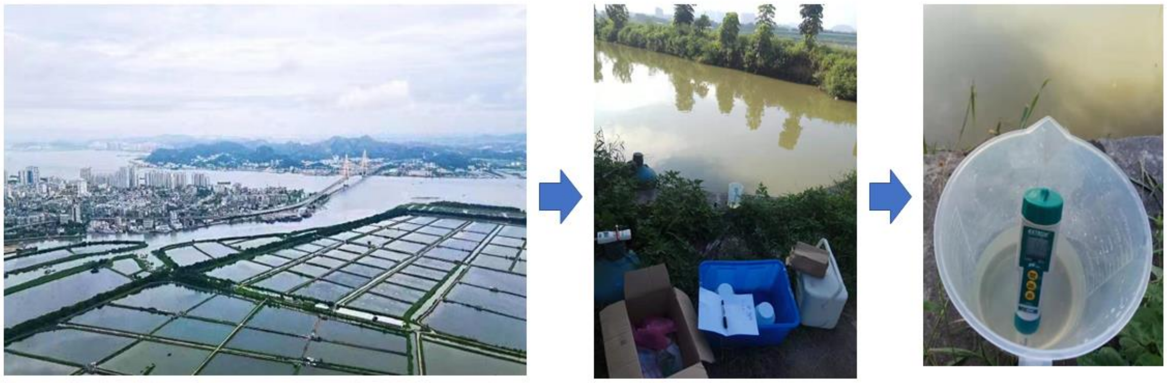

Supplementary Figure S2: Actual figures of sampling locations

Supplementary Table S1. Psychochemical parameters of shrimp ponds and surrounding sea water

**Samples**	**pH**	**Temperature (°C)**	**ORP (mv)**	**Salinity** (%)	**COD** (mg/L)	**Ammonium NH_4** (mg/L)
P_1-S_1	8.1	28.9	86mv	0.5	45.4	2.67
P_1-S_2	7.8	29.5	84	0.5	45.4	2.67
P_1-S_3	8.0	28.8	87	0.5	45.4	2.67
P_2-S_1	8.2	28.3	62	0.8	35.3	2.49
P_2-S_2	8.1	28.8	63	0.8	35.3	2.49
P_2-S_3	7.8	28.6	64	0.8	35.3	2.49
P_3-S_1	7.8	29.0	60	0.9	35.3	2.34
P_3-S_2	7.6	29.2	61	0.9	35.3	2.34
P_3-S_3	7.7	28.6	59	0.9	35.3	2.34
P_4-S_1	8.5	28.6	38	1	38.7	3.77
P_4-S_2	8.2	28.3	39	0.9	38.7	3.77
P_4-S_3	8.1	28.8	41	0.9	38.7	3.77
S-S_1	7.2	29.7	71	1	55.5	2.29
S-S_2	7.1	29.3	69	1	55.5	2.29
S-S_3	7.2	29.5	72	1	55.5	2.29

Supplementary Table S2. BLAST results of the isolated stains

**Top-hit taxon**	**Top-hit strain**	**Similarity**	**Top-hit taxonomy**
Aeromonas veronii	CECT 4257	99.16 %	Bacteria, Proteobacteria, Gamma proteobacteria, Aeromonadales, Aeromonadaceae, *Aeromonas*
Acinetobacter lactucae	NRRL B-41902	99.84 %	Bacteria, Proteobacteria, Gammaproteobacteria, Moraxellales, Moraxellaceae, *Acinetobacter*
Exiguobacterium profundum	10 C	99.37 %	Bacteria, Firmicutes, Bacilli, Bacillales, Exiguobacterium_f, *Exiguobacterium*
Halobacillus dabanensis	D-8	99.49 %	Bacteria, Firmicutes, Bacilli, Bacillales, Bacillaceae, *Halobacillus*
Halobacillus marinus	KG W1	99.79 %	Bacteria, Firmicutes, Bacilli, Bacillales, Bacillaceae, *Halobacillus*
Algoriphagus sanaruensis	M8 -2	99.15 %	Bacteria, Bacteroidetes, Cytophagia, Cytophagales,Cyclobacteriaceae, *Algoriphagus*
Vibrio cholerae	CECT 514	98.99 %	Bacteria, Proteobacteria, Gammaproteobacteria, Vibrionales,Vibrionaceae, *Vibrio*
Bacillus horikoshii	M-8	99.79 %	Bacteria, Firmicutes, Bacilli, Bacillales, Bacillaceae, *Sutcliffiella*
Algoriphagus taiwanensis	CC-PR-82	99.47 %	Bacteria, Bacteroidetes, Cytophagia, Cytophagales,Cyclobacteriaceae, Algoriphagus
Bacillus vietnamensis	15-1	98.94 %	Bacteria, Firmicutes, Bacilli, Bacillales, Bacillaceae, *Bacillus*
Penaeicola halotolerans/ Cyclobacteriaceae bacterium	LMIT005	91.81 %	Bacteria, Bacteroidetes, Cytophagia, Cytophagales,Cyclobacteriaceae, *Algoriphagus*
Nocardioides aestuarii	JC2056	99.05 %	Bacteria, Actinobacteria, Actinomycetia, Propionibacteriales, Nocardioidaceae, *Nocardioides*
Metabacillus indicus	LMG 22858	99.26 %	Bacteria, Firmicutes, Bacilli, Bacillales, Bacillaceae, *Metabacillus*

Erythrobacter alti	KMU-34	97.16 %	Bacteria, Proteobacteria, Alphaproteobacteria, Sphingomonadales, Erythrobacteraceae, *Aurantiacibacter*
Shewanella decolorationis	S12	99.86 %	Bacteria, Proteobacteria, Gammaproteobacteria, Alteromonadales, Shewanellaceae, *Shewanella*

Supplementary Table S3. Details of selected sampling area

**Sampling spot**	**4 ponds and 1 sea water**
Location & Latitude	(23.36 °N, 116.66 °E)
Distance between ponds and surrounding sea water	Pond-Pond: Adjacent to each at a distance of 115-140 meters.<br>Pond-surrounding Sea water: connected via canal from sea water at distance of 970 meters.
Length, width, and depth	200, 60, and 1.5 m
Water sample collection depth	28 cm
Date	September 2019
Antibiotics applied	Sarafloxacin, florfenicol, enrofloxacin, and oxytetracycline
Shrimp	*Litopenaeus vannamei*
